# Potential of the Compounds from *Bixa orellana* Purified Annatto Oil and Its Granules (Chronic^®^) against Dyslipidemia and Inflammatory Diseases: In Silico Studies with Geranylgeraniol and Tocotrienols

**DOI:** 10.3390/molecules27051584

**Published:** 2022-02-28

**Authors:** Mateus Alves Batista, Abrahão Victor Tavares de Lima Teixeira dos Santos, Aline Lopes do Nascimento, Luiz Fernando Moreira, Indira Ramos Senna Souza, Heitor Ribeiro da Silva, Arlindo César Matias Pereira, Lorane Izabel da Silva Hage-Melim, José Carlos Tavares Carvalho

**Affiliations:** 1 Laboratory of Pharmaceutical and Medicinal Chemistry (PharMedChem), Federal University of Amapá, Amapá, Macapá 68902-280, Brazil; mateusbatista.mab@gmail.com (M.A.B.); lorane@unifap.br (L.I.d.S.H.-M.); 2 Laboratory of Drugs Research, Biology and Healthy Sciences Department, Pharmacy Faculty, Federal University of Amapá, Rod. JK, km 02, Amapá, Macapá 68902-280, Brazil; abrahaolima28@gmail.com (A.V.T.d.L.T.d.S.); ali.nascimento99@gmail.com (A.L.d.N.); contatoprofluizmoreira@gmail.com (L.F.M.); heitor_ribeiro_silva@hotmail.com (H.R.d.S.); 3 Diamantina Chapada Regional Hospital, Avenida Francisco Costa, 350-468, Vasco Filho, Bahia, Seabra 46900-000, Brazil; sennaindira@gmail.com; 4 Faculty of Pharmaceutical Sciences of Ribeirão Preto, University of São Paulo (USP), São Paulo, Ribeirão Preto 05508-000, Brazil; arlindo.matias@usp.br

**Keywords:** *Bixa orellana*, oil, inflammatory process, geranylgeraniol, tocotrienol

## Abstract

Some significant compounds present in annatto are geranylgeraniol and tocotrienols. These compounds have beneficial effects against hyperlipidemia and chronic diseases, where oxidative stress and inflammation are present, but the exact mechanism of action of such activities is still a subject of research. This study aimed to evaluate possible mechanisms of action that could be underlying the activities of these molecules. For this, in silico approaches such as ligand topology (PASS and SEA servers) and molecular docking with the software GOLD were used. Additionally, we screened some pharmacokinetic and toxicological parameters using the servers PreADMET, SwissADME, and ProTox-II. The results corroborate the antidyslipidemia and anti-inflammatory activities of geranylgeraniol and tocotrienols. Notably, some new mechanisms of action were predicted to be potentially underlying the activities of these compounds, including inhibition of squalene monooxygenase, lanosterol synthase, and phospholipase A_2_. These results give new insight into new mechanisms of action involved in these molecules from annatto and Chronic^®^.

## 1. Introduction

Lipid disorders, such as dyslipidemia, constitute a significant concern among the overall population and researchers due to their role in hyperlipidemia, hypertension, atherosclerosis, and even insulin resistance. Such aggravation is caused by increased levels of total cholesterol and low-density lipoprotein (LDL) and decreased levels of high-density lipoprotein, which together raise the risk of cardiovascular diseases and metabolic abnormalities [1,2,3,4].

*Bixa orellana* is the plant species known as “annatto” and “achiote”. This species is studied for some health issues, including inflammation-related conditions and dyslipidemias [5,6,7]. Such health benefits can be at least partly due to the presence of tocotrienols and geranylgeraniol from its composition. Tocotrienols are unsaturated forms of vitamin E known for anti-inflammatory, antioxidant, and lipid-lowering activities, which are higher than those from tocopherols—their saturated counterparts, also parts of the vitamin E group [8,9]. In turn, geranylgeraniol is an intermediate in the biosynthesis of cholesterol, and it is believed to regulate the activity of 3-hydroxy-3-methylglutaryl-CoA (HMG-Coa) reductase negatively.

Both tocotrienols and geranylgeraniol are research subjects due to their biological activities, including cardioprotective and neuroprotective effects, hypolipidemic activity, metabolic disorder prevention, and antitumoral activity [10,11,12]. A fundamental approach in the process of drug discovery is pharmaceutical chemistry. A research can be more efficient through pharmaceutical chemistry by decreasing the necessary time, funds, and number of animals needed.

Some of the parameters often screened in potential new drugs through this approach are biological activity prediction, pharmacokinetic profile, and toxicological potential [13,14]. Hence, by using pharmaceutical chemistry tools, the purpose of this study was to evaluate the pharmaceutical potential of tocotrienols and geranylgeraniol for their main biological activities and possible mechanisms of action. This perspective could hint at safer medications compared with the standard ones.

## 2. Results and Discussion

### 2.1. Molecules’ Structure Obtention and Biological Activity Prediction

**Figure 1:**
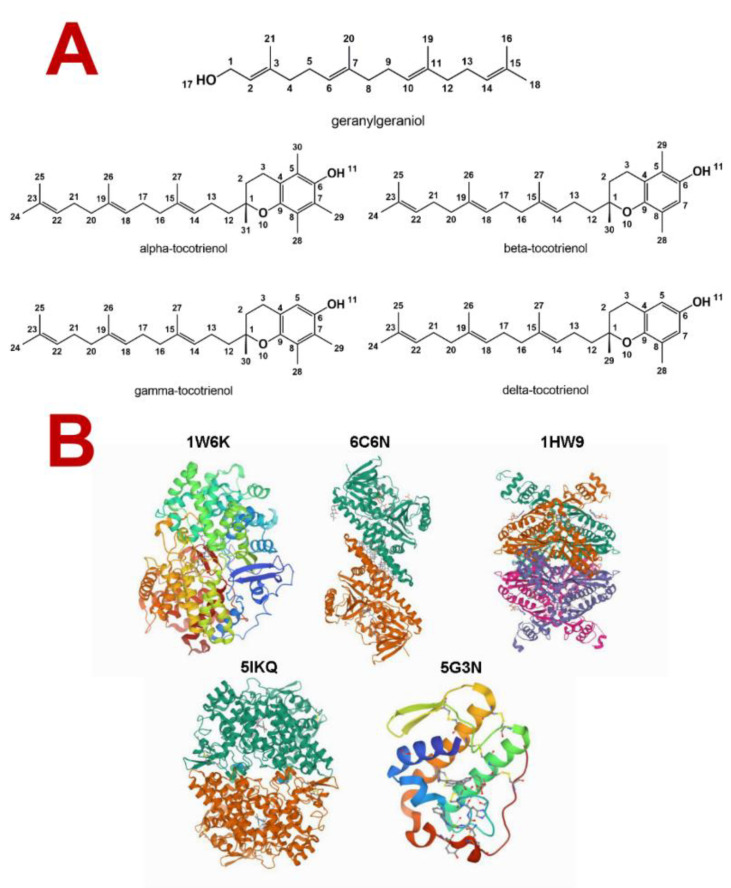
(**A**) Molecular structure of geranylgeraniol and tocotrienols. (**B**) Targets used in the docking simulation with their respective PDB ID. 1W6K: lanosterol synthase complexed with lanosterol; 6C6N: squalene monooxygenase complexed with FAD and CPMPD-4; 1HW9: HMG-CoA reductase complexed with simvastatin; 5IKQ: cyclooxygenase-2 complexed with meclofenamic acid; 5G3N: secreted phospholipase A_2_ complexed with the inhibitor Azd2716.

Tocotrienols and geranylgeraniol are molecules well described and studied in the literature [15,16]. Their structures were obtained from the PubChem database (Figure 1A) and then assessed for possible biological activities and mechanisms of action using the server PASS (prediction of activity spectra for substances) [17,18,19,20].

Geranylgeraniol had a high probability of activity (Pa) values (>0.7) for the following activities: mucous membrane protection (0.953), lipid metabolism regulation (0.885), TNF expression inhibitor (0.840), antiulcerative (0.770), and antineoplastic (0.743). Still notably, the hypolipidemic activity Pa was 0.686, and antihypercholesterolemic Pa was 0.570, both higher than the probability of inactivity (Pi) (0.015 for both).

Tocotrienols also had significant Pa values for lipid peroxidase inhibition (from 0.941 to 0.989), antioxidant activity (from 0.913 to 0.973), anti-inflammatory activity (from 0.813 to 0.866), antihypercholesterolemic activity (from 0.803 to 0.962), cholesterol synthesis inhibition (from 0.663 to 0.702), among other related activities (Table 1). There are some variations among the isomers, but the class consistently shows high Pa’s tendency to improve the blood lipid profile. It is important to notice that in annatto, the most abundant isomer is δ, according to some authors, which can be up to 90% of the isomer composition [21].

To corroborate the results predicted by PASS, we further assessed these compounds through SEA (similarity ensemble approach) [22,23]. The outputs of this server are shown in Table 2. Geranylgeraniol had significant values (*p*-value < 10^−10^ or max Tanimoto coefficient (MaxTC) > 0.6) for squalene monooxygenase (*p*-value = 2.6 × 10^−27^, MaxTC = 0.65) and lanosterol synthase (*p*-value = 4 × 10^−19^, MaxTC = 0.40) interaction probability based on similarity with other compounds. Additionally, the server predicted significant interaction probability with phospholipase A_2_ (*p*-value = 7.3 × 10^−18^, MaxTC = 0.3). Tocotrienols had a lower degree of similarity with compounds able to interact with these targets compared with geranylgeraniol; however, the values were still in a considerable range. For squalene monooxygenase interaction, *p*-values ranged from 2.2 × 10^−08^ to 8.6 × 10^−09^, and MaxTC ranged from 0.30 to 0.31; for lanosterol synthase, *p*-values varied from 1.2 × 10^−06^ to 2.0 × 10^−08^, and MaxTC varied from 0.30 to 0.31. Finally, for phospholipase A_2_, *p*-values varied from 3 × 10^−09^ to 6.6 × 10^−09^, and MaxTC varied from 0.3 to 0.31.

The outputs predicted by PASS and SEA collectively point to these molecules’ tendency to improve the blood lipid profile. However, while in PASS, the most favorable results were achieved by tocotrienols, the highest similarity outputs suggesting that biological action was achieved by geranylgeraniol in SEA. In SEA, the probability of squalene monooxygenase and lanosterol synthase inhibition by tocotrienols was not negligible but was still not high enough. However, it should be kept in mind that these two mechanisms of action are not the only ones that could decrease cholesterol biosynthesis and improve the blood lipid profile. In fact, tocotrienols have been reported to inhibit the mevalonate pathway of HMG-CoA reductase, a pivotal player in cholesterol biosynthesis [24]. While geranylgeraniol was predicted to inhibit lanosterol synthase and monooxygenase in SEA, this was not predicted by PASS. This divergence between the servers could be a negative indicator of these targets, or it could be due to differences in the servers’ training sets, which could give different outcomes.

Reports support a potential role in improving blood lipid profile by geranylgeraniol. For instance, just like tocotrienols, this molecule was shown to decrease HMG-CoA reductase activity [25,26]. Considering the role of this enzyme in cholesterol biosynthesis, this could be a mechanism in which geranylgeraniol exerts its action. Our group reported that the treatment with geranylgeraniol improved blood lipid parameters; however, the molecule was not administrated alone but with tocotrienols [8]. Altogether, the in silico prediction with its known mechanism of action justifies future studies with this molecule alone in treating blood dyslipidemia in vivo.

As mentioned previously, it is believed that this activity may be at least in part due to HMG-CoA reductase inhibition based on previous studies. However, we sought to assess whether more mechanisms were underlying such activity. Hence, molecular docking was performed with the most promising targets.

### 2.2. Molecular Docking

Molecular docking is a powerful tool in computation chemistry that allows researchers to assess the molecular interactions’ type and intensity between a ligand and a target biomolecule within an active site [27]. A total of five macromolecular targets acquired from PDB were used in GOLD without the cocrystalized ligands (Figure 1B). Three of them are involved in cholesterol metabolism (OSC, SQLE, and HMGR), and two are directly involved in inflammation (PLA_2_ and COX-2).

Lanosterol synthase (a.k.a. oxidosqualene cyclase (OSC)) is a membrane-bound protein responsible for synthesizing steroids in mammals. Its cyclization reaction forms lanosterol. Due to its role in the synthesis of steroids, this protein is considered a target to hypolipidemic drugs [28]. When complexed with OSC, lanosterol forms hydrogen bonds with the amino acid residues Trp581 and Asp455 [29].

In the docking performed with OSC, geranylgeraniol and tocotrienol had relevant interactions with the receptors’ active-site amino acid residues. The details of such interactions are shown in Table 3, including the interaction type, distances, and docking scores.

**Figure 2:**
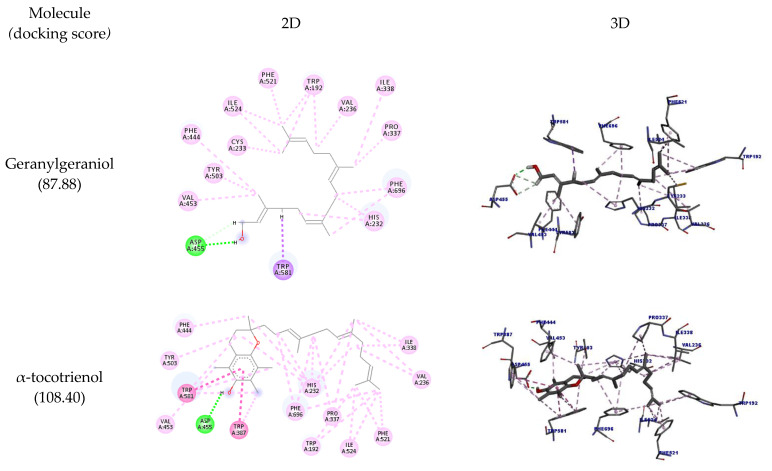

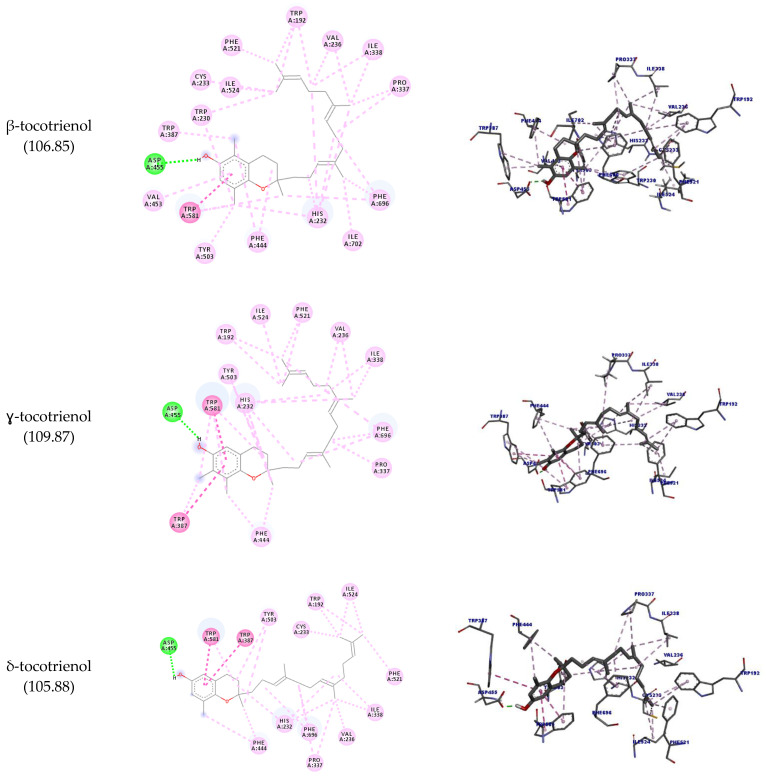
Two-dimensional and three-dimensional representations of the best docking poses calculated by GOLD with OSC (PDB ID: 1W6K). Pictures produced with Discovery Studio.

In Figure 2, it is possible to observe the docking pose in two and three dimensions. It is observed that all molecules could interact with the amino acid residues Asp455 and Trp581 (hydrogen bonds), the same amino acids that can interact with the inhibitor of the enzyme Ro 48-8071, which is considered a structural base for the design of OSC inhibitors. However, the inhibitor performs hydrophobic interactions with Trp581 instead of hydrogen bonds [29].

Like Ro 48-8071, the molecules could also interact with the residues Trp192 and Phe521, indicating that they can potentially inhibit this enzyme. β-tocotrienol could interact with all the residues mentioned so far plus Trp230, thus performing the same interactions of Ro 48-8071.

**Figure 3:**
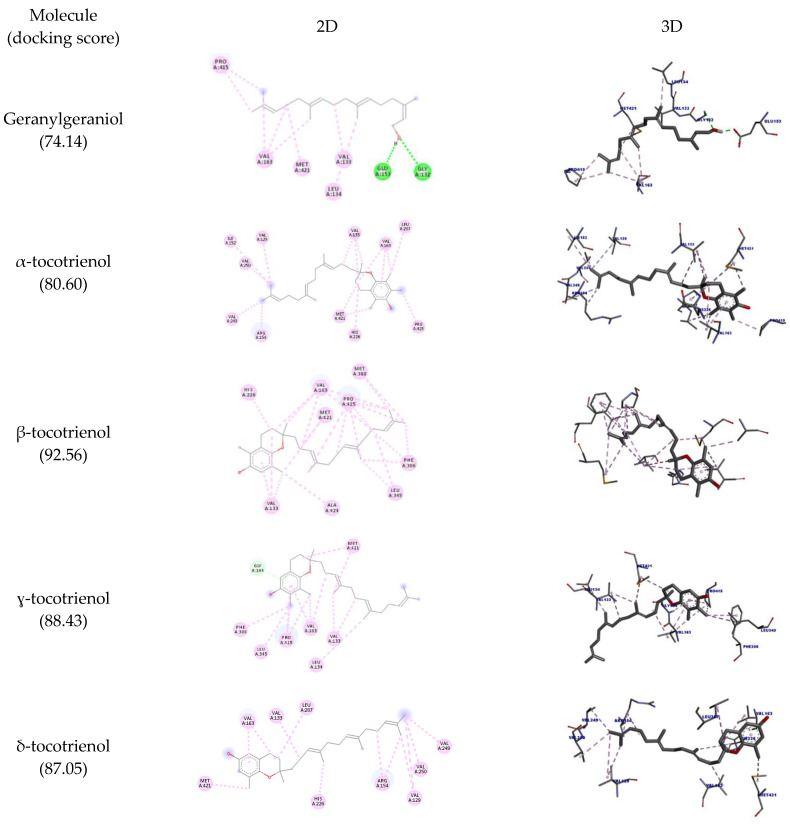
Two-dimensional and three-dimensional representations of the best docking poses calculated by GOLD with SQLE (PDB ID: 6C6N). Pictures produced with Discovery Studio.

Squalene monooxygenase (a.k.a. squalene epoxidase (SQLE)) is the second limiting enzyme in cholesterol biosynthesis accountable to catalyze the conversion of squalene to 2,3(S)-oxidosqualene using flavin adenosine dinucleotide (FAD) as a coenzyme. SQLE inhibition is considered a possible mechanism in treating hypercholesterolemia, fungal infections, and some types of cancer [30]. The docking data with SQLE are shown in Table 4, and the docking poses are depicted in Figure 3.

The aromatic groups of the ligand complexed with SQLE (PDB ID: 6C6N) perform nonpolar interactions with the amino acid residues Asp166, Tyr195, Ala322, Leu333, Tyr335, Pro415, Leu416, and Gly418 [30]. Of these residues, only Pro415 could interact with all the molecules tested (hydrophobic interaction) except for δ-tocotrienol. However, other interactions were observed with different amino acid residues. β-tocotrienol was the compound with more interactions with Pro415 (six hydrophobic interactions) and had the highest docking score (92.56).

**Figure 4:**
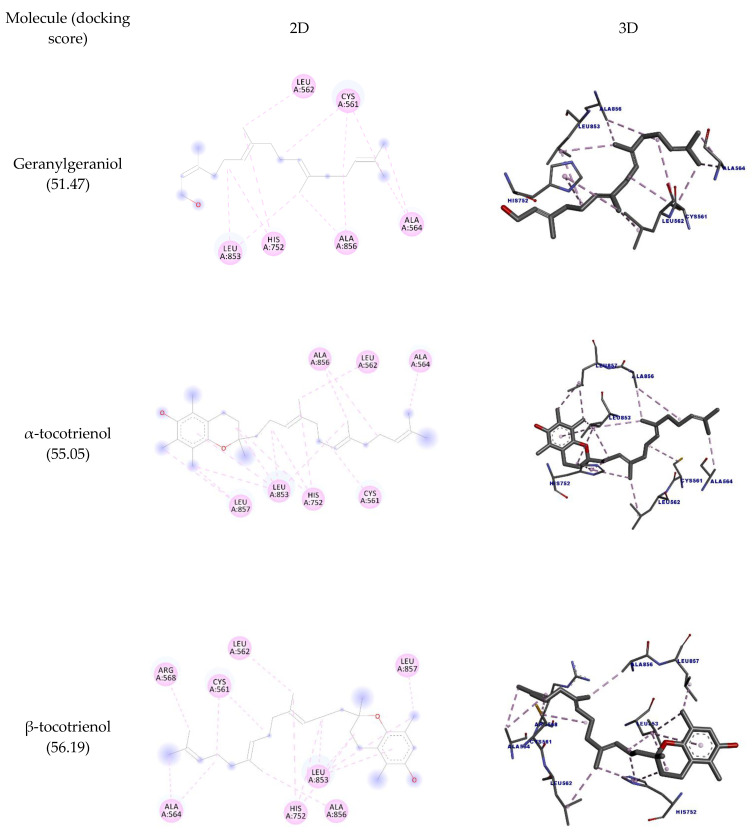

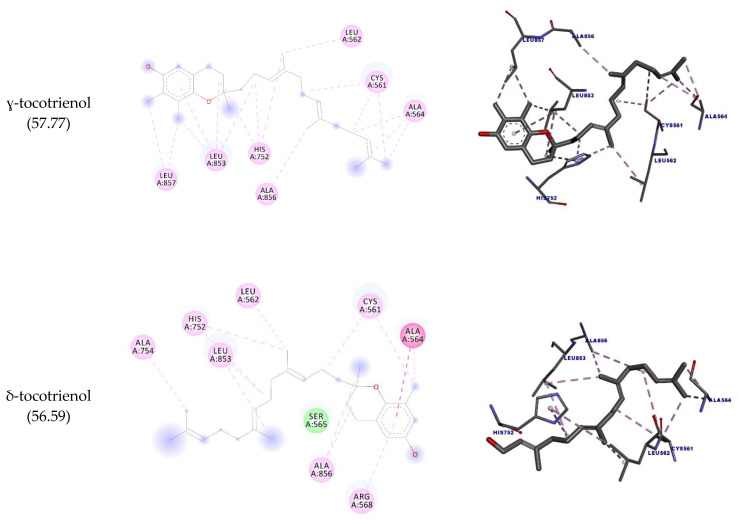
Two-dimensional and three-dimensional representations of the best docking poses calculated by GOLD with HMG-COA reductase. Pictures produced with Discovery Studio.

It is believed that one of the main targets for the hypocholesterolemic activity of tocotrienols is HMG-CoA reductase. This enzyme catalyzes the rate-limiting step in cholesterol biosynthesis [31] and is also targeted by statins, although these molecules inhibit its activity in a different way [8]. As mentioned, there are some reports of HMGR inhibition by geranylgeraniol as well. Here we sought to discover whether the inhibition of these molecules could involve direct binding to HMGR. The docking interactions are detailed in Table 5 and depicted in Figure 4. The results show that the molecules interacted with the amino acid residues Leu562, Leu853, Ala856, and Leu857 through hydrophobic interactions. It is observed that the highest number of interactions and docking score were obtained by γ-tocotrienol (17 interactions; 57.77 docking score), while geranylgeraniol had the lowest (12 and 51.47, respectively).

Inflammation is tightly associated with lipid and metabolic disturbances [32,33,34]. According to the results predicted by PASS and SEA, geranylgeraniol and tocotrienols may also decrease inflammation. In accordance with our results, it has been reported that geranylgeraniol suppresses the expression of interleukin-1 receptor-associated kinase-1 (IRAK1) and tumor necrosis factor receptor-associated factor 6 (TRAF6), consequently preventing NF-κB excessive activation in LPS-induced inflammatory response in THP-1 cells. In addition, tocotrienols are thought to exert their effects also in part by decreasing the inflammatory cascade [35,36,37,38,39,40].

Since SEA predicted the interaction of all the molecules with phospholipase A_2_, we performed a docking with this enzyme. We also performed docking with COX-2 because it is a common target for anti-inflammatory compounds (such as the NSAIDs).

**Figure 5:**
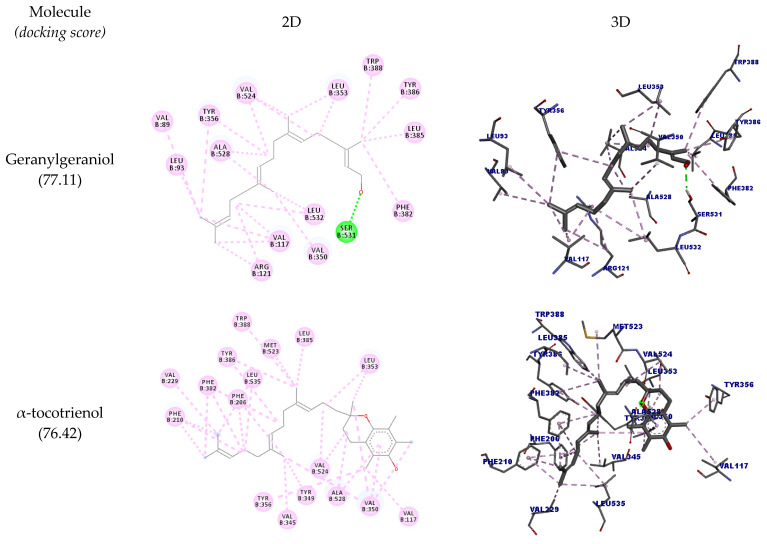

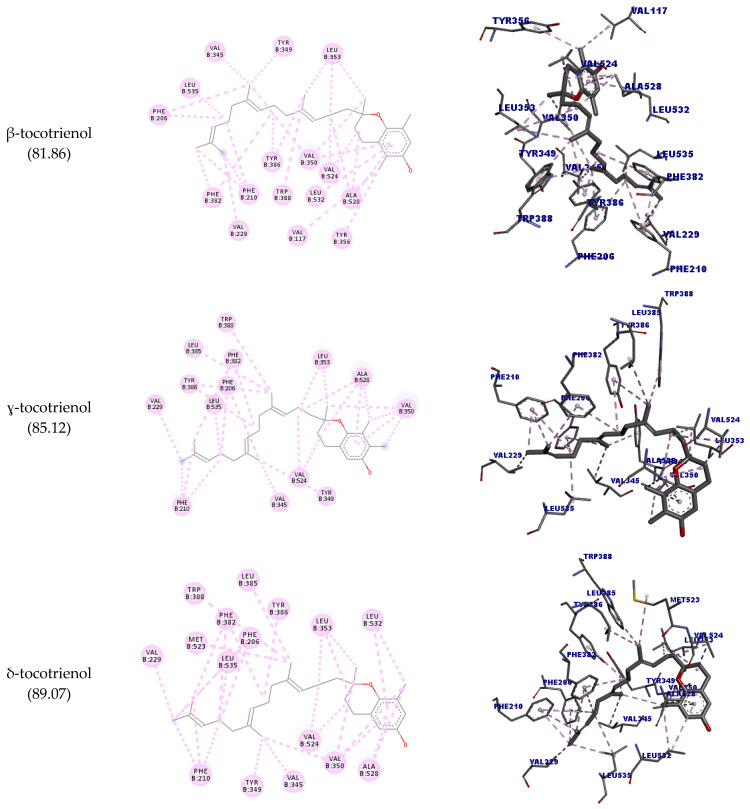
Two-dimensional and three-dimensional representations of the best docking poses calculated by GOLD with COX-2 (PDB ID: 5IKQ). Pictures produced with Discovery Studio.

COX-2 is an inflammatory enzyme that converts arachidonic acid into prostaglandins, such as prostaglandin H2 [41]. The docking results with COX-2 are shown in Table 6, and the docking poses are depicted in Figure 5. The structure of COX-2 was stored in PDB in a complex with meclofenamic acid, a known inhibitor of this enzyme.

The hydrogen bonds between the inhibitor’s carboxylate and the phenolic oxygen of Tyr385 and Ser530 are considered important interactions for the inhibition of this enzyme [42]. It was observed that all the structures could interact with COX-2, but none of them could interact with the amino acid residues Tyr385 and Ser530. The highest docking score was achieved by δ-tocotrienol (89.07), and the other molecules had good scores as well (>70).

Phospholipase A_2_ is another enzyme involved in the inflammatory response that catalyzes the hydrolysis of two glycerophospholipids and releases two fatty acids and lysophospholipids. The secreted PLA_2_ is involved in the rate-limiting step of eicosanoid biosynthesis by releasing unesterified arachidonic acid from membrane phospholipids [43].

**Figure 6:**
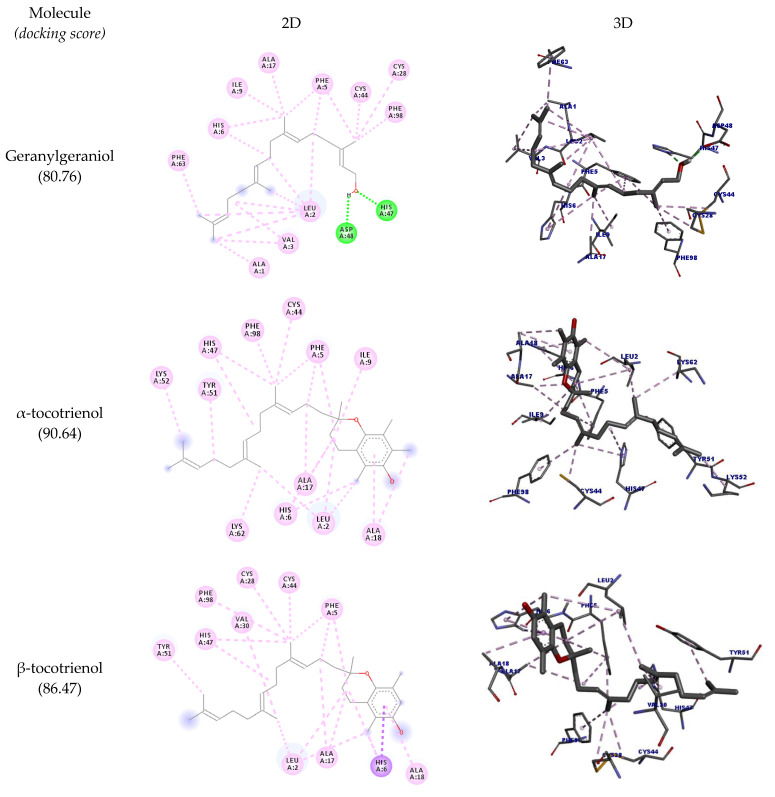

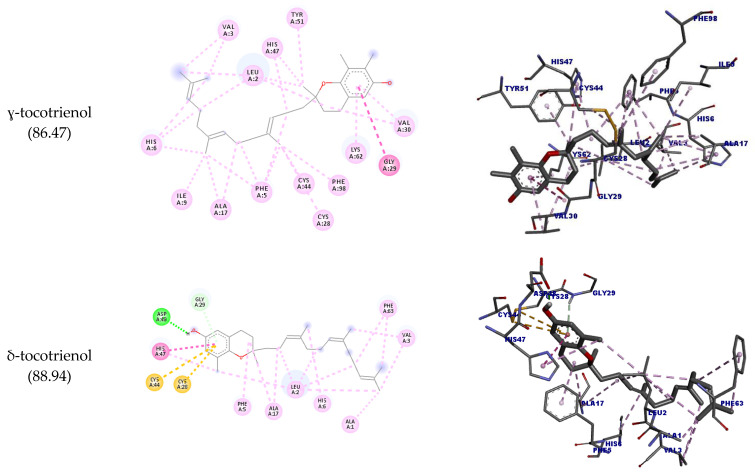
Two-dimensional and three-dimensional representations of the best docking poses calculated by GOLD with PLA_2_ (PDB ID: 5G3N). Pictures produced with Discovery Studio.

Table 7 shows all the interactions of this enzyme with geranylgeraniol and tocotrienols, and the best docking poses are depicted in Figure 6. The results show that all molecules interacted with the amino acid residue His47; except for α-tocotrienol, all molecules could interact with Cys28 as well. Most of the molecules assessed could interact with PLA_2_’s hydrophobic pocket (Leu2, Phe5, His5, Ile9, Ala17, Ala8, Gly22), suggesting this enzyme’s potential inhibition. The highest docking score was achieved by α-tocotrienol (90.64).

In the docking studies, it was observed that geranylgeraniol could interact with all the targets assessed. For OSC, SQLE, and PLA_2_, these interactions were similar to their corresponding crystalized inhibitors, corroborating the predictions by SEA and suggesting a potential hypocholesterolemic and anti-inflammatory activity. Tocotrienols also could interact with the assessed enzymes; notably, β-tocotrienol had an interesting interaction profile with OSC, similar to Ro 48-8071. As regards SQLE, δ-tocotrienol could not interact with the target’s active site amino acid residues, while all others could interact with Pro415, specially β-tocotrienol.

Although all molecules could interact with COX-2, none of these interactions are reported in the literature to inhibit this enzyme activity. For PLA_2_, an important interaction that inhibits this enzyme is with the amino acid residues His47 and Cys28. All tocotrienols could interact with His47, and all but α-tocotrienol could interact with Cys28 as well (even though this molecule had the highest docking score).

Collectively, the docking supports the biological activity prediction. The results support the hypocholesterolemic and anti-inflammatory potential for geranylgeraniol and tocotrienols, following previous reports in the literature. Although these activities are not new for these molecules, our results suggest some potential new action mechanism that has not been reported, such as lanosterol synthase inhibition, which is different from HMG-CoA reductase inhibition.

### 2.3. Pharmacokinetic Property Prediction

Despite having a desired biological activity, a compound must effectively reach its therapeutic targets, and for this, the molecule must have a favorable pharmacokinetic profile (absorption, distribution, metabolism, excretion (ADME)). Nowadays, several approaches are available to predict ADME data from compounds [44]. The servers PreADMET and SwissADME were used to indicate such activities based on the compounds’ structures. The data are shown in Table 8**.**

In PreADMET outputs, %HIA represents the human intestinal absorption, which, as the name suggests, refers to the amount of the molecule that is absorbed. HIA is important because most drugs are administered orally and hence need to be absorbed in satisfactory amounts in the gastrointestinal tract [45]. The server PreADMET considers that good drug candidates should have a %HIA of at least 70%. Hence, all the molecules had a great degree of intestinal absorption with %HIA > 97%, and geranylgeraniol had 100%.

SwissADME bases the gastrointestinal absorption and blood–brain barrier permeation on a different model called BOILED-Egg (brain or intestinal estimated permeation method) [46,47]. In this distinct model, geranylgeraniol but not tocotrienols were predicted to be highly permeant to the GI tract due to their high Lop P.

A popular model to assess drug absorption in drug discovery is using Caco-2 or MDCK cells as test systems. PreADMET can predict the molecular permeation in these cells by comparing the molecules from those of its database. According to the server, <4 nm/s represents low permeation, values from 4 to 70 nm/s have intermediate permeation, and values above that represent high permeation. For MDCK, values below 25 represent low permeability, values from 25 to 500 represent intermediate permeation, and values above 500 represent high permeation [48,49].

All molecules assessed had intermediate absorption values in Caco-2 cells, while in MDCK, only geranylgeraniol and δ-tocotrienol had intermediate absorption values, and the others had low values. Overall, geranylgeraniol had superior results to tocotrienols. Among tocotrienols, α-tocotrienol had the highest absorption values (Table 8).

For PreADMET, good drug candidates must have <90% of blood protein binding (BPB) because the molecules should be free to be able to interact with their biological targets [50]. In our prediction, the molecules had an unfavorable BPB profile (higher than 90%). Another distribution parameter assessed was the interaction with P-glycoprotein (P-gp) calculated by SwissADME. This macromolecule is responsible for hampering the intracellular accumulation of potentially toxic compounds and removing them from the CNS through the blood–brain barrier as well [51]. The server predicted that tocotrienols could interact with these targets while geranylgeraniol could not.

Both servers give outputs about blood–brain barrier (BBB) permeation and, hence, have potential to reach the CNS. However, the results are in disagreement. According to PreADMET, compounds with Cbrain/Cblood values higher than 2.0 can cross the BBB, and all the molecules had high values, while in Swiss ADME, which uses the BOILED-Egg model, the molecules were predicted not to cross the BBB. However, these molecules probably cross the BBB according to in vivo data of tocotrienols and other vitamins E in SNC disorders [52,53]. The pharmacokinetics of tocotrienols have been reported in patients with favorable results and safety profiles [54,55].

### 2.4. Toxicological Property Prediction

The toxicological prediction from geranylgeraniol and tocotrienols were assessed with PreADMET and ProTox-II. This online server is accessible and can help screen possible toxicities from compounds [56]. The prediction outputs are shown in Table 9.

All the molecules were predicted to be nonmutagenic in bacteria and nonhepatotoxic, cardiotoxic, immunotoxic, or cytotoxic. The predicted median lethal doses were high, especially for geranylgeraniol. ProTox-II classifies the molecules according to the predicted toxicity from 1 to 6, in which higher values represent less toxic compounds. The highest value was achieved for geranylgeraniol (5), while tocotrienols were classified as 4.

## 3. Materials and Methods

### 3.1. Molecules Studied

This study used the major molecules found in the purified annatto oil (PAO) and its granules (Chronic^®^). The samples were kindly provided by Ages Bioactive Compounds Co. (São Paulo-SP, Brazil). The batch analysis certificate is described as URU200401 (12 March 2020, expiration date: 22 March 2022), composition: bixin (1.7%), tocotrienols (9.59%), and geranylgeraniol (28.32%), as described by Matias Pereira et al. [8].

All structures used were confirmed in the PubChem database (https://pubchem.ncbi.nlm.nih.gov/, accessed on 1 October 2021) (Figure 1A). The molecules were drawn using ChemDraw [56] and optimized using HyperChem through the semiempirical method RM1 [57].

### 3.2. Biological Activities Prediction

The prediction of biological activity was based on analysis of the structure–activity relationship of a training set using the PASS server (prediction of activity spectra for substances; http://www.pharmaexpert.ru/passonline, accessed on 1 October 2021), which can predict 4.130 biological activities in the compounds with an average accuracy of 95%. PASS is based on the naïve Bayes classifier approach and multilevel neighborhoods of atoms descriptors. The predicted activities are given as Pa (probability of being active) or Pi (probability to be inactive). Molecules with a Pa superior to 0.7 are considered promising candidates for the given activity; however, molecules with Pa > 0.4 and Pa > Pi could still be good candidates [17,18,19,20].

In addition, the SEA server (similarity ensemble approach; http://sea.bkslab.org/, accessed on 1 November 2021) was used to assess potential targets of the studied molecules. This server predicts small-molecule activity based on the macromolecular targets they interact with, which is inferred according to topology similarity with other molecules’ fingerprints from its database [22,23]. The server gives the *p*-value as similarity output representing the expected value (E-value) and the max Tanimoto coefficient (MaxTC). In a prediction, the lower the *p*-value, the more significant it is, evidencing that the prediction is less likely to be by chance; ideally, a prediction should be <10^−10^ to be highly significant, while a *p*-value > 1 is considered insignificant. A MaxTC is considered highly significant when the value is >0.6, and insubstantial when <0.3 [22,58].

### 3.3. Molecular Docking

The docking was performed using the software GOLD (Genetic Optimization for Ligand Docking [59]) using biological targets acquired from Protein Data Bank [60]. A total of five targets were selected: the human lanosterol synthase (an oxidosqualene cyclase (OSC)) complexed with lanosterol, human squalene epoxidase (a.k.a. squalene monooxygenase (SQLE)) complexed with FAD and CPMPD-4, human HMG-CoA reductase (HMGR) complexed with simvastatin, secreted phospholipase A_2_ (sPLA_2_) complexed with the inhibitor Azd2716, and human cyclooxygenase-2 (COX-2) complexed with meclofenamic acid (Figure 1B). All the cocrystalized ligands were removed to perform the docking.

Before the dockings, validation was performed for each target by calculating the root mean square deviation (RMSD), which is the root mean square distance of nonhydrogen atoms of the ligand from the crystal structure and their corresponding docked pose. All the crystallized targets had RMSD < 2 Å and considered the upper limit of satisfactory docking [61]. Other parameters assessed were the docking sphere radius and x, y, and z coordinates (Table 10).

Cocrystallized ligands, ions, and water molecules were removed from the crystallographic structures to perform the docking. Additionally, hydrogens were added to the ligands, and their atomic charge was calculated using HyperChem, as described in [62].

### 3.4. Pharmacokinetic Prediction

An in silico ADME (absorption, distribution, metabolism, excretion) prediction was performed using the servers PreADMET (https://preadmet.bmdrc.kr/, accessed on 1 November 2021) and SwissADME (http://www.swissadme.ch, accessed on 1 November 2021). These servers can calculate the physicochemical and pharmacokinetic properties of molecules, including human intestinal absorption, Caco-2 cell and MDCK permeability, percentage of plasma protein binding, blood–brain barrier penetration, glycoprotein P interaction, metabolism by P450 cytochromes, among others [46,48,63].

### 3.5. Toxicological Prediction

The toxicological prediction was performed using ProTox-II. This server can predict different toxicity parameters, such as acute toxicity, organ-specific toxicity, cytotoxicity, carcinogenicity, and immunotoxicity [64].

## 4. Conclusions

The biological activity results follow what is reported in the literature, mainly for the antioxidant, anti-inflammatory, and antidyslipidemia potential of geranylgeraniol and tocotrienols. The molecular docking corroborated the predicted activities of the servers. Notably, the in silico data presented another mechanism of action that could be involved in the activity of this molecule, which is inhibition of squalene monooxygenase and lanosterol synthase, which will need to be confirmed in vitro.

These in silico data corroborate the use of these molecules against lipid disorders, coronary disease due to cholesterol accumulation, and several chronic diseases in which oxidative stress and inflammatory cascade have a role. Geranylgeraniol and tocotrienols are major molecules from *Bixa orellana* and Chronic^®^. The results also point to a good pharmacokinetic profile for these molecules and a good safety profile, according to previously reported experimental data.

## Figures and Tables

**Table 1:** Biological activity prediction of the compounds according to the PASS server.

Molecule	Pa	Pi	Activity Prediction
Geranylgeraniol	0.953	0.003	Mucous membrane protection
	0.885	0.004	Lipid metabolism regulation
	0.840	0.003	TNF inhibitor
	0.770	0.004	Antiulcerative
	0.743	0.049	Antineoplastic
	0.686	0.015	Hypolipidemic
	0.636	0.007	NF kappa B regulator
	0.643	0.024	Anti-inflammatory
	0.570	0.015	Antihypercholesterolemic
	0.549	0.005	Antioxidant
	0.538	0.03	Cholesterol antagonist
	0.498	0.019	Antineoplastic
	0.437	0.007	Cholesterol synthesis inhibitor
α-tocotrienol	0.989	0.001	Lipid peroxidase inhibitor
	0.973	0.002	Antioxidant
	0.962	0.002	Antihypercholesterolemic
	0.900	0.005	Treatment of acute neural disorders
	0.892	0.005	Cerebral anti-ischemic
	0.866	0.005	Anti-inflammatory
	0.863	0.003	Peroxidase inhibitor
	0.763	0.005	Hepatoprotector
	0.753	0.034	Mucous membrane protection
	0.713	0.008	Cholesterol antagonist
	0.702	0.001	Cholesterol synthesis inhibition
	0.685	0.003	NOS_2_ expression inhibition
	0.621	0.009	Antineoplastic (breast cancer)
	0.456	0.033	NF kappa B inhibitor
	0.426	0.031	Atherosclerosis treatment
	0.435	0.046	TNF inhibitor
	0.397	0.044	Antipsoriasis
	0.255	0.017	Phospholipase A_2_ inhibition
β-tocotrienol	0.957	0.002	Lipid peroxidase inhibition
	0.951	0.002	Antioxidant
	0.951	0.002	Antihypercholesterolemic
	0.881	0.004	Hypolipidemic
	0.835	0.005	Anti-inflammatory
	0.812	0.005	Anticarcinogenic
	0.787	0.004	Antiulcerative
	0.744	0.002	NOS_2_ expression inhibition
	0.738	0.040	Mucous membrane protection
	0.692	0.001	Cholesterol synthesis inhibition
	0.714	0.026	Cerebral anti-ischemic
	0.685	0.008	Hepatoprotector
	0.648	0.035	Antineoplastic
	0.602	0.019	Cholesterol antagonist
	0.475	0.027	Antipsoriasis
	0.481	0.034	TNF inhibitor
	0.355	0.010	NF kappa B inhibitor
	0.271	0.026	Lipoprotein disorder treatment
	0.198	0.025	Phospholipase A_2_ inhibition
ɣ-tocotrienol	0.977	0.002	Lipid peroxidase inhibition
	0.953	0.002	Antioxidant
	0.944	0.002	Antihypercholesterolemic
	0.882	0.004	Hypolipidemic
	0.846	0.005	Anti-inflammatory
	0.811	0.005	Anticarcinogenic
	0.776	0.017	Cerebral anti-ischemic
	0.762	0.004	Antiulcerative
	0.686	0.001	Cholesterol synthesis inhibitor
	0.682	0.008	Hepatoprotector
	0.719	0.008	Mucous membrane protection
	0.683	0.003	NOS_2_ expression inhibition
	0.593	0.011	Antineoplastic (breast cancer)
	0.452	0.041	TNF inhibitor
	0.464	0.061	Lipid metabolism inhibitor
	0.402	0.043	Antipsoriasis
	0.271	0.014	NF kappa B inhibitor
	0.230	0.016	Phospholipase A_2_ inhibition
	0.280	0.091	Atherosclerosis treatment
δ-tocotrienol	0.941	0.002	Lipid peroxidase inhibition
	0.913	0.003	Antioxidant
	0.813	0.006	Anti-inflammatory
	0.803	0.005	Antihypercholesterolemic
	0.791	0.008	Hypolipidemic
	0.789	0.022	Mucous membrane protection
	0.745	0.002	NOS_2_ expression inhibition
	0.683	0.005	Antiulcerative
	0.663	0.001	Cholesterol synthesis inhibition
	0.650	0.011	Anticarcinogenic
	0.642	0.036	Antineoplastic
	0.589	0.013	Hepatoprotector
	0.522	0.025	TNF inhibition
	0.512	0.027	Antithrombotic
	0.515	0.041	Lipid metabolism regulation
	0.458	0.03	Antipsoriasis
	0.444	0.147	Cerebral anti-ischemic
	0.385	0.007	NF kappa B inhibitor
	0.224	0.038	Lipoprotein disorder regulator
	0.201	0.024	Phospholipase A_2_ inhibitor

**Table 2:** Prediction outputs of the molecules assessed with ligands from the SEA server.

Molecule	Target	*p*-Value	Max TC
Geranylgeraniol	Squalene monooxygenase	2.641 × 10^−27^	0.65
	Lanosterol synthase	4.01 × 10^−19^	0.40
	Phospholipase A_2_	7.305 × 10^−18^	0.31
	Protein-S-isoprenylcysteine O-methyltransferase	1.703 × 10^−65^	0.53
	Geranylgeranyl pyrophosphate synthase	1.409 × 10^−61^	0.50
	Transient receptor potential cation channel subfamily V member 2	1.407 × 10^−49^	0.38
	Transient receptor potential cation channel subfamily A member 1	6.621 × 10^−40^	0.40
	Protein farnesyltransferase subunit beta	2.46 × 10^−10^	0.53
	Protein farnesyltransferase/geranylgeranyltransferase type 1 subunit alpha	9.389 × 10^−10^	0.53
α-tocotrienol	Alpha-tocopherol transfer protein	4.81 × 10^−63^	0.52
	PH domain leucine-rich repeat-containing protein phosphatase 1	4.19 × 10^−15^	0.34
	Phospholipase A_2_	6.66 × 10^−09^	0.30
	Squalene monooxygenase	8.61 × 10^−09^	0.31
	DNA polymerase lambda	1.39 × 10^−08^	0.29
	Lanosterol synthase	2.04 × 10^−08^	0.31
	Geranylgeranyl pyrophosphate synthase	9.35 × 10^−04^	0.29
β-tocotrienol	PH domain leucine-rich repeat-containing protein phosphatase 1	6.38 × 10^−51^	0.39
	Alpha-tocopherol transfer protein	1.62 × 10^−21^	0.36
	DNA Polymerase lambda	1.69 × 10^−20^	0.33
	Phospholipase A_2_	3 × 10^−09^	0.31
	Squalene monooxygenase	2.23 × 10^−08^	0.3
	Lanosterol synthase	1.2 × 10^−06^	0.3
γ-tocotrienol	PH domain leucine-rich repeat-containing protein phosphatase 1	3.13 × 10^−65^	0.57
	DNA polymerase lambda	1.69 × 10^−20^	0.33
	Alpha-tocopherol transfer protein	1.61 × 10^−09^	0.32
	Phospholipase A_2_	3 × 10^−09^	0.31
	Squalene monooxygenase	2.23 × 10^−08^	0.3
	Lanosterol synthase	9.42 × 10^−07^	0.31
δ-tocotrienol	PH domain leucine-rich repeat-containing protein phosphatase 1	7.59 × 10^−50^	0.39
	DNA polymerase lambda	8.97 × 10^−20^	0.32
	Phospholipase A_2_	3 × 10^−09^	0.31
	Squalene monooxygenase	2.23 × 10^−08^	0.3
	Lanosterol synthase	9.42 × 10^−07^	0.31
	Hypoxia-inducible factor 1-alpha	5.31 × 10^−06^	0.36

**Table 3:** Docking interactions of the molecules with OSC.

Molecule	Amino Acid	Ligand Atom	Category	Types	Distance (Å)	Score
Geranylgeraniol	**A:ASP455**	H25	Hydrogen bond	Conventional hydrogen bond	2.08	87.88
					2.65	
					2.81	
	**A:TRP581**	H28	Hydrophobic	Pi-sigma	2.87	
	A:VAL236	Ligand		Alkyl	5.36	
	A:VAL453	C14			3.94	
	A:PRO337	C16			4.90	
	A:ILE338				5.25	
	A:ILE524	C20			3.70	
	A:CYS233	C21			4.53	
	A:ILE524				4.57	
	**A:TRP192**	Ligand		Pi-alkyl	5.12	
		C20			5.40	
		C21			4.63	
	A:HIS232	Ligand			4.69	
					4.24	
	A:PHE444	C14			4.92	
	A:TYR503				4.76	
	**A:PHE521**	C20			3.93	
	A:PHE696	Ligand			3.91	
		C15			4.10	
α-tocotrienol	**A:ASP455**	H39	Hydrogen bond	Conventional hydrogen bond	1.86	108.40
	**A:TRP581**	Ligand	Hydrophobic	Pi-pi stackedPi-pi T-shaped	4.38	
					4.26	
	A:TRP387				5.60	
	A:VAL236			Alkyl	5.28	
	A:PRO337				5.33	
	A:VAL453	C11			4.26	
	A:ILE338	Ligand			5.18	
	A:VAL236	C26			5.03	
	A:PRO337				4.99	
	A:ILE338				4.29	
	A:ILE524	C30			3.55	
		C31			5.29	
	**A:TRP192**	C30		Pi-alkyl	4.71	
		C31			5.27	
	A:HIS232	ligand			4.84	
					5.07	
		C16			5.24	
		ligand			5.31	
					4.69	
		C26			4.93	
	A:TRP387	C13			4.96	
					4.30	
	A:PHE444	C16			4.46	
	A:TYR503	Ligand			5.11	
	**A:PHE521**	C30			4.58	
		C31			3.62	
	**A:TRP581**	C13			5.00	
		Ligand			4.64	
		C14			5.26	
	A:PHE696	ligand			4.76	
					4.55	
		C31			5.39	
β-tocotrienol	**A:ASP455**	H39	Hydrogen bond	Conventional hydrogen bond	2.17	106.85
	**A:TRP581**	Ligand	Hydrophobic	Pi-pi stacked	4.80	
					4.27	
	A:VAL236			Alkyl	4.48	
	A:PRO337				4.72	
	A:ILE702	C20			4.41	
	A:ILE338	Ligand			5.15	
		C25			5.33	
	A:PRO337				4.40	
	A:ILE338				4.09	
	A:CYS233	C29			4.57	
	**A:ILE524**				3.70	
	**A:TRP192**	Ligand		Pi-alkyl	5.17	
		C29			5.46	
		C30			5.08	
	**A:TRP230**	C13			5.26	
					4.85	
		C29			5.01	
	A:HIS232	C13			4.70	
		Ligand			4.15	
					5.49	
					5.23	
		C25			5.33	
	A:TRP387	C11			4.72	
					3.73	
	A:PHE444	Ligand			4.13	
		C11			4.51	
	A:TYR503	C13			4.59	
	**A:PHE521**	C30			3.69	
	**A:TRP581**	C13			4.02	
		C15			4.97	
	A:PHE696				5.20	
		Ligand			5.19	
		C20			3.83	
	A:VAL453	Ligand			5.32	
ɣ-tocotrienol	**A:ASP455**	H36	Hydrogen bond	Conventional hydrogen bond	1.83	109.87
	**A:TRP581**	Ligand	Hydrophobic	Pi-pi stacked	4.31	
	**A:TRP581**				4.18	
	A:TRP387				5.69	
	A:VAL236			Alkyl	5.38	
	A:PRO337				5.46	
	A:ILE338				5.15	
	A:VAL236				5.07	
	A:ILE338	C24			4.37	
	A:ILE524				3.63	
	**A:TRP192**	C28		Pi-alkyl	4.59	
		C29			5.20	
	A:HIS232	Ligand			4.75	
					5.09	
		C14			5.07	
		Ligand			4.95	
		C24			4.77	
	A:TRP387	C30			4.90	
					4.17	
	A:PHE444	C12			5.14	
		C14			4.59	
	A:TYR503	Ligand			5.01	
		C14			5.30	
	**A:PHE521**	C28			4.18	
		C29			3.59	
	**A:TRP581**	C30			4.96	
		Ligand			4.61	
		C12			5.42	
	A:PHE696	Ligand			4.80	
					4.34	
		C29			5.23	
δ-tocotrienol	**A:ASP455**	H36	Hydrogen bond	Conventional hydrogen bond	1.66	105.88
	**A:TRP581**	Ligand	Hydrophobic	Pi-pi stacked	4.28	
					4.15	
	A:TRP387			Pi-pi T-shaped	5.80	
	A:PRO337			Alkyl	5.47	
	A:ILE338				5.00	
	A:VAL236	C24			5.46	
	A:PRO337				4.58	
	A:ILE338	C28			4.28	
	A:CYS233				4.29	
	A:ILE524	C29			3.72	
		C28			4.78	
	**A:TRP192**	C29		Pi-alkyl	5.01	
		Ligand			5.31	
	A:HIS232				4.85	
		C24			4.95	
		C12			5.03	
	A:PHE444	C14			4.48	
		Ligand			3.76	
	A:TYR503	C14			5.22	
		C29			5.42	
	**A:PHE521**	Ligand			3.92	
	**A:TRP581**				5.18	
	A:PHE696				5.18	
		H55			4.32	

**Table 4:** Docking interactions of the molecules with SQLE.

Molecule	Amino Acid	Ligand Atom	Category	Types	Distance (Å)	Score
Geranylgeraniol	A:GLY132	O24	Hydrogen bond	Conventional hydrogen bond	2.83	74.14
	A:GLU153	H55			1.70	
	A:VAL133	Ligand	Hydrophobic	Alkyl	4.06	
	A:VAL163				5.13	
	A:MET421				5.04	
	A:LEU134	C15			4.41	
	A:VAL163	C16			4.56	
		C20			4.57	
	**A:PRO415**				4.05	
		C21			4.27	
α-tocotrienol	A:VAL133	Ligand	Hydrophobic	Alkyl	4.33	80.60
					5.27	
	A:VAL163				5.21	
	A:MET421				4.42	
		C11			4.04	
	**A:PRO415**	C13			3.96	
	A:VAL163	C14			3.71	
	A:LEU287				4.19	
	A:VAL133	C16			4.70	
	A:VAL129	C30			4.93	
	A:ILE152				5.02	
	A:VAL250				4.05	
	A:ARG154	C31			4.14	
	A:VAL249				4.20	
	A:HIS226	Ligand		Pi-alkyl	4.96	
	A:VAL163				4.36	
β-tocotrienol	A:VAL133	Ligand	Hydrophobic	Alkyl	5.10	92.56
	A:VAL163				4.65	
					5.00	
					4.55	
	**A:PRO415**				4.92	
					4.72	
	A:ALA424	C13			3.84	
	A:VAL133				4.39	
	A:MET421				5.43	
		Ligand			4.71	
	A:VAL163	C15			4.73	
	**A:PRO415**	C20			4.76	
	A:LEU345	Ligand			5.16	
		C25			4.75	
	**A:PRO415**				4.32	
	A:VAL163	C29			4.76	
	A:MET388				4.47	
	**A:PRO415**				4.60	
	A:MET388	C30			4.90	
	**A:PRO415**				4.11	
	A:HIS226	Ligand		Pi-alkyl	4.95	
	A:PHE306				5.25	
		C25			4.84	
		C30			4.56	
	A:VAL133	Ligand			3.93	
ɣ-tocotrienol	GLY164	ligand	Hydrogen bond	Pi-donor hydrogen bond	2.67	88.43
	VAL133		Hydrophobic	Alkyl	4.71	
	VAL163				4.67	
	MET421				4.68	
	VAL163	C12			3.82	
	**PRO415**				4.40	
	VAL133	C19			3.89	
	MET421				4.79	
	LEU134	C24			5.06	
	LEU345	C30			4.32	
	**PRO415**				4.60	
	PHE306			Pi-alkyl	4.73	
	VAL163	Ligand			5.45	
	**PRO415**				4.94	
δ-tocotrienol	VAL133	Ligand	Hydrophobic	Alkyl	4.37	87.05
	ARG154				4.86	
	VAL163				5.06	
	LEU287				4.66	
	MET421	C12			4.62	
	VAL129	C28			5.47	
	VAL249				4.49	
	VAL250				4.51	
	VAL129	C29			5.18	
	ARG154				5.13	
	HIS226	C19		Pi-alkyl	5.47	
	VAL163	Ligand			3.93	

**Table 5:** Docking interactions of the molecules with HMG-CoA reductase.

Molecule	Amino Acid	Ligand Atom	Category	Types	Distance (Å)	Score
**Geranylgeraniol**	CYS561	C9	Hydrophobic	Alkyl	4.49	51.47
		C17			5.46	
	ALA564	C20			3.30	
		C21			3.71	
	**ALA856**	C17			4.86	
		C16			3.49	
	**LEU853**	C5			4.21	
	**LEU562**	C15			3.83	
	**LEU853**	C16			4.26	
	CYS561	C20			3.86	
	HIS752	C5		Pi-alkyl	4.36	
		C15			4.97	
**α-tocotrienol**	CYS561	C22	Hydrophobic	Alkyl	4.18	55.05
	ALA564	C31			3.51	
	**ALA856**	C27			5.19	
		C26			3.30	
	**LEU853**	C9			5.05	
	**LEU857**	C13			4.29	
	**LEU853**	C14			4.63	
	**LEU857**				4.30	
	**LEU853**	C17			4.16	
	**LEU562**	C21			3.90	
	**LEU853**	C26			4.73	
	HIS752	C9		Pi-alkyl	5.03	
		C17			4.37	
		C21			4.95	
	**LEU853**	Anel Ar.			5.15	
**β-tocotrienol**	CYS561	C21	Hydrophobic	Alkyl	4.31	56.19
		C26			4.40	
	ALA564				4.37	
		C29			4.15	
	**ALA856**	C25			3.63	
	**LEU853**	C9			5.33	
		C13			4.56	
	**LEU857**				4.20	
	**LEU853**	C16			4.19	
	**LEU562**	C20			3.92	
	ARG568	C30			3.97	
	HIS752	C9			5.32	
		C16		Pi-alkyl	4.53	
		C20			4.71	
	**LEU853**	Anel Ar.			5.35	
**ɣ-tocotrienol**	CYS561	C20	Hydrophobic	Alkyl	4.15	57.77
		C25			4.97	
	ALA564				4.87	
		C28			3.34	
		C29			3.70	
	**ALA856**	C24			3.32	
	**LEU853**	C9			4.93	
		C12			4.68	
	**LEU857**	C12			4.41	
	**LEU853**	C15			4.11	
	**LEU562**	C19			4.04	
	CYS561	C28			3.75	
	**LEU857**	C30			4.24	
	HIS752	C9		Pi-alkyl	4.88	
		C15			4.40	
		C19			5.06	
	**LEU853**	Anel Ar.			5.01	
**δ-tocotrienol**	ALA564	Anel Ar.	Hydrophobic	Amide-pi stacked	4.02	56.59
	CYS561	C15		Alkyl	4.18	
	ALA564	C12			3.33	
	ALA754	C29			4.03	
	**ALA856**	C9			4.26	
		C14			4.43	
	CYS561	C12			3.32	
	**LEU853**	C20			4.24	
	**LEU562**	C19			4.24	
	**LEU853**	C24			4.74	
	HIS752	C20		Pi-alkyl	4.27	
		C19			4.56	
	ALA564	Anel Ar.			4.21	
	ARG568				5.40	

**Table 6:** Docking interactions of the molecules with COX-2.

Molecule	Amino Acid	Ligand Atom	Category	Types	Distance (Å)	Score
Geranylgeraniol	B:SER531	O24	Hydrogen bond	Conventional hydrogen bond	2.16	77.11
	B:VAL117	Ligand	Hydrophobic	Alkyl	3.86	
	B:ARG121				5.14	
	B:VAL524				4.13	
					4.59	
	B:ALA528				3.99	
		C16			3.39	
	B:LEU353	Ligand			5.04	
	B:LEU532				5.28	
	B:LEU385	C14			5.05	
	B:LEU353	C15			4.18	
	B:VAL524				3.88	
	B:VAL350	C16			4.57	
	B:LEU532				4.40	
	B:VAL89	C20			5.46	
	B:LEU93				4.92	
	B:VAL117				4.43	
		C21			3.45	
	B:ARG121				4.48	
	B:TYR356	Ligand		Pi-alkyl	5.30	
		C20			5.09	
	B:PHE382	C14			5.48	
	B:TYR386				4.31	
	B:TRP388				4.91	
α-tocotrienol	B:VAL524	Ligand	Hydrophobic	Alkyl	3.65	76.42
					4.60	
	B:ALA528				3.85	
	B:VAL117	C11			5.03	
	B:VAL350	C13			3.68	
	B:LEU353	Ligand			4.79	
	B:VAL350	C16			5.10	
	B:LEU353				4.14	
	B:LEU385	C21			4.79	
	B:MET523				4.91	
	B:LEU535	Ligand			4.79	
	B:VAL345	C26			4.76	
	B:VAL350				5.09	
	B:VAL229	C30			4.50	
	B:LEU535				4.93	
	B:PHE206	Ligand		Pi-alkyl	4.82	
		C26			5.43	
	B:PHE210	Ligand			4.89	
		C30			4.27	
		C31			4.24	
	B:TYR349	C26			4.50	
	B:TYR356	C11			4.00	
	B:PHE382	Ligand			4.43	
					5.15	
		C31			4.60	
	B:TYR386	Ligand			4.49	
		C21			4.91	
	B:TRP388				4.99	
	B:VAL350	Ligand			4.04	
	B:ALA528				3.86	
β-tocotrienol	B:VAL524	Ligand	Hydrophobic	Alkyl	3.84	81.86
					4.58	
	B:ALA528				4.17	
					4.48	
	B:VAL117	C11			5.10	
	B:LEU353	Ligand			5.34	
	B:VAL350	C15			4.49	
	B:LEU353				3.92	
		C20			4.72	
	B:LEU535	Ligand			4.26	
	B:VAL345	C25			4.39	
	B:VAL350				5.49	
	B:VAL229	C29			4.89	
	B:PHE206	Ligand		Pi-alkyl	4.50	
		C25			4.87	
	B:PHE210	Ligand			4.84	
		C29			3.85	
		C30			4.64	
	B:TYR349	C25			4.77	
	B:TYR356	C11			4.36	
	B:PHE382	Ligand			4.85	
		C30			4.53	
	B:TYR386	Ligand			4.57	
		C20			4.74	
	B:TRP388				4.55	
	B:VAL350	Ligand			4.32	
	B:ALA528				3.74	
	B:LEU532				4.74	
ɣ-tocotrienol	B:VAL524	Ligand	Hydrophobic	Alkyl	3.62	85.12
					4.85	
	B:ALA528				4.50	
		C12			3.69	
		Ligand			4.81	
	B:VAL350	C12			4.19	
	B:LEU353	Ligand			4.95	
	B:VAL350	C14			4.83	
	B:LEU353				3.85	
	B:LEU385	C19			4.93	
	B:LEU535	Ligand			4.36	
	B:VAL345	C24			4.59	
	B:VAL229	C28			4.90	
	B:VAL350	C30			4.34	
	B:PHE206	Ligand		Pi-alkyl	4.67	
		C24			4.89	
	B:PHE210	Ligand			4.82	
		C28			3.84	
		C29			4.55	
	B:TYR349	C24			4.63	
	B:PHE382	Ligand			4.60	
		C29			4.45	
	B:TYR386	Ligand			4.54	
		C19			4.58	
	B:TRP388	C19			4.90	
	B:VAL350	Ligand			4.42	
	B:ALA528				4.37	
δ-tocotrienol	B:VAL524	Ligand	Hydrophobic	Alkyl	3.81	89.07
					4.52	
	B:ALA528	C12			3.63	
	B:LEU353	Ligand			5.45	
	B:VAL350	C12			4.34	
	B:LEU532				4.66	
	B:LEU353	Ligand			4.84	
	B:VAL350	C14			4.32	
	B:LEU353				3.75	
	B:LEU385	C19			4.74	
	B:MET523				4.65	
	B:LEU535	Ligand			4.89	
	B:VAL345	C24			5.08	
	B:VAL350				4.84	
	B:VAL229	C28			4.45	
	B:LEU535				4.71	
	B:PHE206	Ligand		Pi-alkyl	4.84	
		C28			5.37	
	B:PHE210	Ligand			5.04	
		C28			4.26	
		C29			4.39	
	B:TYR349	C24			4.51	
	B:PHE382	Ligand			4.81	
					5.13	
		C29			4.65	
	B:TYR386	Ligand			4.52	
	B:TRP388	C19			5.15	
	B:VAL350	Ligand			4.84	
	B:ALA528				3.55	
	B:LEU532				5.25	

**Table 7:** Docking interactions of the molecules with PLA_2_.

Molecule	Amino Acid	Ligand Atom	Category	Types	Distance (Å)	Score
Geranylgeraniol	**HIS47**	O24	Hydrogen bond	Conventional hydrogen bond	1.61	80.76
	ASP48	H55			1.97	
	ALA1	C21	Hydrophobic	Alkyl	3.79	
	VAL3	Ligand			4.87	
	**ALA17**	C15			3.70	
	**LEU2**	Ligand			5.21	
					4.34	
					4.89	
	**CYS28**	C14			4.24	
	CYS44				4.33	
	**ILE9**	C15			4.98	
	**LEU2**	C16			3.94	
		C20			4.71	
		C21			5.30	
	VAL3				4.51	
	**PHE5**	Ligand		Pi-alkyl	4.84	
		C14			5.20	
		C15			4.31	
	**HIS6**	Ligand			4.98	
		C15			5.02	
	PHE63	C20			4.26	
	PHE98	C14			4.85	
α-tocotrienol	**ALA17**	Ligand	Hydrophobic	Alkyl	3.99	90.64
					5.28	
		C13			4.21	
	**LEU2**	Ligand			4.98	
	**ILE9**				5.49	
	**LEU2**	C11			4.89	
	CYS44	C21			3.95	
	**LEU2**	C26			4.39	
	LYS62				4.68	
	LYS52	C30			3.99	
	**PHE5**	Ligand		Pi-alkyl	4.58	
	**PHE5**				4.92	
		C21			4.59	
	**HIS6**	Ligand			5.40	
		C21			4.71	
	**HIS47**	Ligand			4.84	
		C21			4.78	
	TYR51	Ligand			4.82	
	PHE98	C21			4.51	
	**ALA18**	Ligand			4.67	
β-tocotrienol	**HIS6**	Ligand	Hydrophobic	Pi-sigma	2.89	86.47
	**ALA17**			Alkyl	4.15	
					5.17	
	**LEU2**				4.76	
		C11			5.48	
	**CYS28**	C20			4.48	
	CYS44				3.99	
	**LEU2**	C25			4.67	
	VAL30				4.90	
	**PHE5**	Ligand		Pi-alkyl	5.02	
					4.80	
		C20			4.96	
	**HIS6**	Ligand			5.03	
		C11			4.63	
	**HIS47**	Ligand			4.58	
		C20			5.06	
	TYR51	C30			4.52	
	PHE98	C20			5.11	
	**ALA18**	Ligand			4.50	
ɣ-tocotrienol	GLY29	Ligand	Hydrophobic	Amide-pi stacked	4.93	88.94
	**ALA17**			Alkyl	4.79	
		C24			3.57	
	VAL30	Ligand			5.15	
	**LEU2**				5.21	
		C14			4.57	
	**CYS28**	C19			4.35	
	CYS44				4.29	
	**LEU2**	Ligand			3.82	
	**ILE9**	C24			4.82	
	**LEU2**	C28			5.24	
	VAL3				4.48	
		C29			4.61	
	**PHE5**	Ligand		Pi-alkyl	5.18	
					5.39	
		C19			5.11	
		C24			4.28	
	**HIS6**	Ligand			5.43	
		C24			4.96	
		C29			4.33	
	**HIS47**	Ligand			4.47	
		C14			4.72	
	TYR51				4.12	
	PHE98	C19			5.09	
	VAL30	Ligand			4.31	
	LYS62				4.8	
δ-tocotrienol	ASP48	H36	Hydrogen bond	Conventional hydrogen bond	1.71	87.82
	GLY29	Ligand		Pi-donor hydrogen bond	2.93	
	**CYS28**		Other	Pi-sulfur	5.93	
	CYS44				4.86	
	**HIS47**		Hydrophobic	Pi-pi T-shaped	4.78	
	ALA1	C29		Alkyl	3.27	
	**ALA17**	Ligand			4.90	
					4.89	
	**LEU2**	C12			4.11	
		Ligand			4.23	
					4.79	
					4.36	
	VAL3	C24			4.77	
	**LEU2**	C29			4.43	
	VAL3				4.34	
	**PHE5**	Ligand		Pi-alkyl	4.52	
	**HIS6**	C19			4.64	
	**HIS47**	C12			5.20	
	PHE63	Ligand			4.95	
		C28			4.69	

**Table 8:** ADME prediction by PreADMET and SwissADME.

Molecule	PreADMET							
	Absorption			Distribution		Absorption	Distribution	
	%HIA	Caco-2 (nm/sec)	MDCK (nm/sec)	BPB%	BBB (C_brain/_C_blood_)	GI absorption	BBB	P-gp
Geranylgeraniol	100	37.1	62.05	100	17.58	High	No	No
α-tocotrienol	97.91	29.13	21.78	100	19.21	Low	No	Yes
β-tocotrienol	97.9	27.94	24.31	100	19.01	Low	No	Yes
γ-tocotrienol	97.9	27.94	24.31	100	18.99	Low	No	Yes
δ-tocotrienol	97.89	26.83	27.42	100	18.83	Low	No	Yes

**Table 9:** Toxicity prediction in ProTox-II.

Molecule	Toxicity Class	Predicted DL_50_	Toxicity Type	Prediction	Probability
Geranylgeraniol	5	5000 mg/kg	Hepatotoxicity	Inactive	0.79
			Carcinogenicity	Inactive	0.76
			Immunotoxicity	Inactive	0.99
			Mutagenicity	Inactive	0.97
			Cytotoxicity	Inactive	0.85
α-tocotrienol	4	500 mg/kg	Hepatotoxicity	Inactive	0.93
			Carcinogenicity	Inactive	0.77
			Immunotoxicity	Inactive	0.89
			Mutagenicity	Inactive	0.92
			Cytotoxicity	Inactive	0.87
β-tocotrienol	4	500 mg/kg	Hepatotoxicity	Inactive	0.93
			Carcinogenicity	Inactive	0.77
			Immunotoxicity	Inactive	0.79
			Mutagenicity	Inactive	0.92
			Cytotoxicity	Inactive	0.87
γ-tocotrienol	4	500 mg/kg	Hepatotoxicity	Inactive	0.93
			Carcinogenicity	Inactive	0.77
			Immunotoxicity	Inactive	0.61
			Mutagenicity	Inactive	0.92
			Cytotoxicity	Inactive	0.87
δ-tocotrienol	4	500 mg/kg	Hepatotoxicity	Inactive	0.94
			Carcinogenicity	Inactive	0.79
			Immunotoxicity	Inactive	0.93
			Mutagenicity	Inactive	0.91
			Cytotoxicity	Inactive	0.86

**Table 10:** Docking validation parameters.

Molecule	PDB ID	Resolution (Å)	RMSD (Å)	Docking Radius (Å)	x, y, z Coordinates
Lanosterol synthase (OSC)	1W6K	2.1	0.622	11.49	28.79, 69.02, 8.45
Squalene epoxidase (SQLE)	6C6N	2.3	1.038	15.08	−23.75, 92.76, 63.37
HMG-CoA reductase (HMGR)	1HW9	2.3	1.482	8.41	2.31, −8.29, −9.21
Cyclooxygenase-2 (COX-2)	5IKQ	2.4	0.507	8.867	16.06, 43.11, 60.99
Phospholipase A_2_ (sPLA_2_)	5G3N	1.8	0.507	9.132	7.48, 3.41, −0.16

## Data Availability

Not applicable.

## Sample Availability

The samples are available upon request.
